# Stroke Survivor and Caregiver Perspectives on Seeking Emergency Medical Care

**DOI:** 10.1177/23743735261458028

**Published:** 2026-06-03

**Authors:** Emma N. Johnson, Lindsay E. Stewart, Edward C. Jauch, Jane H. Brice, Mehul D. Patel

**Affiliations:** 1 Department of Emergency Medicine, 2331 University of North Carolina at Chapel Hill, Chapel Hill, NC, USA; 2 Department of Research, 26520 University of North Carolina Health Sciences at MAHEC, Asheville, NC, USA; 3 Department of Emergency Medicine, Duke University, Durham, NC, USA

**Keywords:** emergency medicine, patient experience, patient perspectives/narratives, qualitative methods, medical decision making

## Abstract

Early recognition of acute stroke and activation of emergency medical services (EMS) are critical for timely treatment and improved patient outcomes. We aimed to identify facilitators and barriers to calling 9-1-1 for acute stroke from the perspectives of stroke survivors and caregivers. We conducted 42 semi-structured interviews of stroke survivors and caregivers in North Carolina, recruited from an online platform and stroke support groups. Interviews explored recognition of stroke signs and care-seeking decisions. Interviews were analyzed thematically. Facilitators of timely 9-1-1 calls included prior stroke experience, knowledge of stroke signs, severe/rapid-onset symptoms, and the presence of bystanders. Barriers included perceiving mild symptoms, believing EMS was unnecessary, fear of overreacting, financial concerns, and distrust of healthcare providers. Early recognition and EMS activation were sometimes facilitated by contacting a healthcare professional within the patient’s social network. Public stroke education needs to address common misperceptions about symptom severity, appropriate use of EMS, and ambulance costs.

## Introduction

Early recognition of acute stroke and activation of emergency medical services (EMS) by calling 9-1-1 is a critical first step in the stroke care continuum. Failure to call or delays in calling 9-1-1 can have a detrimental impact on stroke patients by prolonging arrival to the emergency department (ED) and missing the time windows for treatments, such as intravenous thrombolytics and endovascular thrombectomy.^1,2^ Moreover, reperfusion therapies are more effective when administered earlier.^3^ Despite the importance of calling 9-1-1, only 50-60% of stroke patients in the United States arrive by EMS.^4^

Several factors influence the decision to call 9-1-1 for stroke-like symptoms. The severity and sudden onset of symptoms often trigger an immediate call for help.^2,5-7^ Conversely, patients with mild or atypical symptoms may perceive symptoms as unserious and hesitate to seek medical attention.^8-11^ The presence of a bystander, especially a family member or caregiver, significantly increases the likelihood of a 9-1-1 call compared to patients who are alone.^5,8,12^ Prior experience with stroke, either personal or through a relative or friend, can also prompt a quicker response.^5,10^ Lastly, awareness and knowledge of stroke signs and symptoms and the importance of calling 9-1-1 are considered important targets for intervention.^5,7^ However, the results of public campaigns to raise stroke awareness and knowledge have been mixed and have largely not been successful in improving EMS use and patient outcomes on a larger scale.^13^

In addition to patient-specific factors, contextual factors like social determinants of health and geographic location play a significant role in the decision to call 9-1-1 for stroke. In general, higher education, income, and health literacy are positively associated with an individual’s urgency in seeking medical attention.^6,14^ People in rural areas face longer distances to the nearest ED or stroke center and are particularly vulnerable to prolonged prehospital delays.^15^ The role of socioeconomic and environmental conditions in decisions to call 9-1-1 may contribute in part to well-documented stroke disparities.

While prior studies have examined factors of prehospital delay and care seeking behaviors,^1,2,8,10,14,16-23^ our study aims to qualitatively assess patients’ and caregivers’ decision-making processes to better understand experiences and perceptions of acute stroke care. To this end, we aimed to identify facilitators and barriers to acute stroke identification and subsequent emergency medical care seeking from a diverse group of stroke survivors and caregivers.

## Methods

### Study Design & Setting

This qualitative descriptive study assessed perspectives of stroke survivors and caregivers to stroke survivors in North Carolina (NC).^24^ The State of NC is an ideal setting in which to examine the role of patient/bystander perspectives on acute stroke care as one of eight states that comprise the Stroke Belt, a region of the southeastern United States that reports disproportionately higher stroke mortality in comparison to the rest of the country.^1^

Individual, semi-structured interviews were conducted with stroke survivors and caregivers concerning care-seeking behaviors during an acute stroke. Topics covered include stroke history, responses to signs and symptoms, and experiences with seeking emergency medical care. Questions were developed from prior published studies and with input from subject matter experts. See Appendix 1 for interview guides. If the interviewee had multiple stroke experiences, the most recent was discussed. Where the stroke survivor had limited communication, interviews were conducted with the survivor-caregiver dyad. This study was reviewed by the University of North Carolina at Chapel Hill Institutional Review Board and determined to be exempt from further review. This manuscript was prepared following the Standards for Reporting Qualitative Research guidelines to ensure transparent and rigorous reporting.^25^

### Study Population

Participants included individuals who survived a stroke themselves and individuals (“caregivers”) who were either present during a stroke event or cared for the patient after their stroke. A convenience sample was recruited from across NC. Initial recruitment used a university-affiliated, public-facing online platform, supplemented by outreach to stroke support groups, hospital stroke units, rehabilitation centers, and senior centers. Snowball recruitment was also employed, asking enrolled participants to share study information with others who might be eligible and interested. All participants were screened for eligibility and stroke experience before scheduling an interview. About midway through enrollment, targeted efforts were made to increase participants from rural areas.

### Interviews

Individual, semi-structured interviews were completed virtually between June and December 2024. The first 5 interviews were conducted using a cognitive interviewing approach to test question comprehension. Interview guides were revised to their final versions based on participant feedback. See Appendix 1 for the final interview guides. All interviews were included in the data analysis.^26-28^ Two clinical research coordinators (ENJ, LES) with no prior relationship to the participants conducted the interviews. Both interviewers had experience in semi-structured interviewing and background knowledge of acute stroke. The interviews were recorded and transcribed using an audio transcription service.

### Qualitative Data Analysis

An initial codebook was deductively derived from themes explored in the interview guides, including stroke experience, perceptions of symptoms, and care-seeking behaviors and decision-making. The transcripts were initially reviewed and coded by two reviewers (ENJ, SL). A first round of coding was conducted with the initial codebook, after which the reviewers compared and discussed all coded segments until a consensus was reached. The initial codebook and consensus-coded segments were used for an iterative second round of coding in which one reviewer (ENJ) analyzed segments and added subcodes to initial codes that captured care-seeking behaviors and decision-making. The second round of coding continued until no new subcodes were identified, ensuring that code saturation was reached.^29,30^ A third reviewer (LES) reviewed subcoded segments and discussed potential changes with the first reviewer (ENJ). Final subcodes and subcoded segments were determined jointly between both reviewers (ENJ, LES), and any disagreements were discussed until consensus was reached. See Appendix 2 for the final codebook.

Consistent with prior literature, we defined time of stroke onset as the time when the patient or a bystander first noted symptoms or signs.^31^ We defined an “early response” as care sought within 1.5 hours of the onset of stroke signs and symptoms whereas a “late response” took longer to seek care.^18-20^ Seeking care was defined as contacting a medical professional - including a physician, nurse, calling 9-1-1, or another emergency professional – either over the phone or in-person.^18,19^ There is no standard threshold for the time interval from stroke onset to calling 9-1-1. The critical time window for administration of intravenous thrombolytics is 4.5 hours, and clinical benefit is maximized with treatment within 3 hours. Therefore, we defined an “early response” as calling 9-1-1 within 1.5 hours of stroke onset, which logistically gives the patient an opportunity to receive treatment in an optimal time frame. Prior stroke experience was defined as any previous lived direct experience (self, loved one) or indirect experience (acquaintance). The county in which the stroke event occurred was classified as rural or non-rural based on population density. Thematic analysis derived facilitators and barriers to calling 9-1-1, which were compared descriptively by response timing, prior stroke experience, and rurality. Qualitative data analyses were conducted in MAXQDA (Verbi Software, Berlin, Germany).

## Results

### Participant Characteristics

Forty-two participants completed interviews, lasting between 17-69 min with a mean duration of 37 min. Table 1 provides the participant and geographic characteristics of the study.

**Table 1.** Characteristics of 42 Interview Participants

Characteristic	Total (N=42)	Survivor (N=25)	Caregiver (N=17)
Gender			
Female	21	10	11
Male	21	15	6
Race*			
Non-Hispanic Black	5	1	4
Non-Hispanic White	36	23	13
Prefer Not to Answer	1	1	0
Prior Stroke Experience	11	4	7
Alone at Symptom Onset	5	5	N/A
Geographic Location			
Mountains (Western NC)	2	2	0
Piedmont (Central NC)	36	20	16
Coastal Plains (Eastern NC)	4	3	1
Rurality*			
Rural	13	7	6
Urban or Suburban	29	18	11

* A rural county was defined as a population density of 250 people per square miles or less. Densities were calculated by the North Carolina Rural Center with 2020 U.S. Census data.

**Figure 1.**
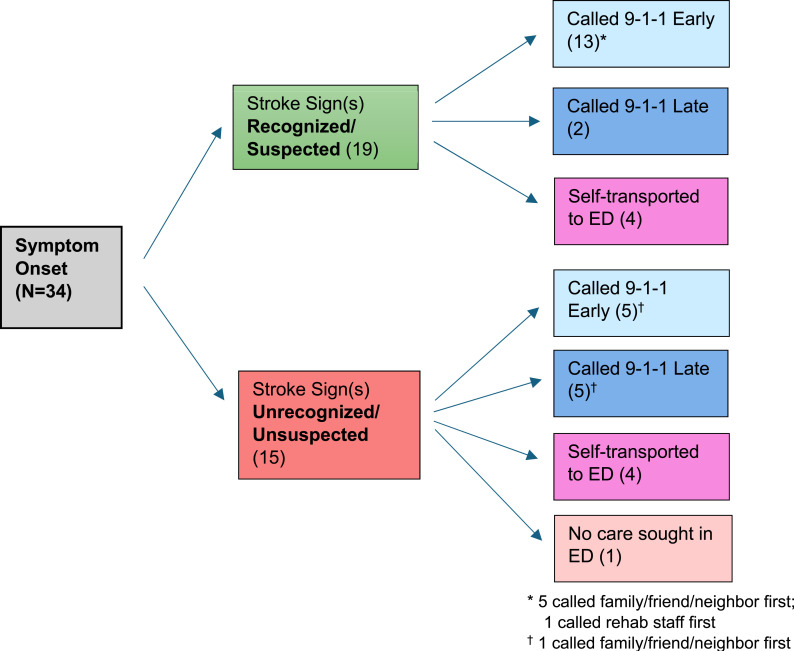
Pathways of stroke recognition and care-seeking responses for 34 stroke events

Because some participants were part of a survivor-caregiver pair, the interviews covered 34 distinct stroke events. Among these, 25 called 9-1-1, 8 self-transported to the ED, and 1 did not seek care in the ED. Of those having called 9-1-1, 18 met our definition for an early response (within 1.5 hours of symptoms) whereas all 7 late responses were beyond 6 hours of symptom onset. Figure 1 illustrates pathways of stroke symptom recognition and these care-seeking responses (more detailed pathways are shown in Figure S1, Supplemental Materials).

Of the 19 events recognized or suspected as stroke signs at the time of onset, most (13, 68%) called 9-1-1 early. When the stroke signs were not recognized or suspected initially, 9-1-1 was less likely to be called early or at all. In our study sample, prior stroke experience, having a caregiver or other bystander present, and non-rural location were all associated with a higher frequency of calling 9-1-1 promptly. However, instances of delayed care-seeking and opting to self-transport instead of calling 9-1-1 were represented in both rural and non-rural settings. For detailed information and participant quotes regarding care-seeking behaviors, see Table 2.

**Table 2.** Facilitators and Barriers to Calling 9-1-1 for Stroke

Theme	Sub-Theme*	Representative Quote
Facilitators	1. Prior stroke	“I think I’m having a stroke, ‘Look at my face,’ and I smiled. Since they were experienced from the year before, we knew what was going on.” –survivor
		“...once I realized I was having a stroke or it was very likely ... because I’ve had friends and family have strokes ... I knew it was really important to get medical help as soon as possible.” –survivor
	2. Bystander influence	“One of the ladies at the vet, her husband had a stroke, so she recognized the symptoms. I think she knew what was going on, but I of course did not know what was going on.” –survivor
		“I would say it was more... of [my daughter’s] decision to go ahead and call [9-1-1] than it was me.” –survivor
	3. Severity of symptoms	“I said, “I can’t move my hand,” and I was then checking to see if I could move my—I said, “I can’t move my leg either.” I was talking to my wife, and she said ‘We’re calling 9-1-1′ She just reached over to her nightstand and grabbed her phone and called 9-1-1. It was almost instantaneous. There was no discussion.” –survivor
		“Well, I recognized that whatever was goin’ on with my right side, something was happening, and I knew I needed emergency care.” –survivor
		“It was definitely apparent that all of his symptoms were presenting a stroke, and we just knew that that was a very serious issue that needed to be dealt with quickly.” –caregiver
	4. Stroke knowledge	“We’re both 59, so we start to pay more attention at the doctors’ offices, those charts that show you, and the billboards that are up sometimes that show you the—and I don’t remember the acronym, but you know, that you look for slurred speech. You look for things with eyes, and you look for—so it was just I knew—I don’t know. I knew because I had been exposed to what stroke symptoms were.” –caregiver
		“My wife, who had taken CPR recently, realized I was havin’ a stroke and called 911 immediately.” –survivor
Barriers	1. Symptoms perceived as not severe	“Like I said, we let him go on for a day or two because we thought it’s another TIA and it’ll go away. The emergency room hadn’t done anything for the TIAs prior, so we just were waiting it out.” –caregiver
		“I figured I would lay down, not eat anything for a few hours, and I’d either wake up in the morning and be fine or wake up sometime later that evening and be fine.” –survivor
	2. Reserve 9-1-1 for life-threatening event	“Yeah, I think I was just very confused, and I had this idea. I don’t know where I got it from, where it’s like you don’t call 911 unless you’re dying.” –caregiver
		“I just didn’t think it was life-threatening, I guess, and so that’s why I just chose to call him [to bring me to the hospital].” –survivor
	3. Concerns of healthcare/EMS costs	“I just thought it would be really expensive and a bad experience.” –survivor
		“You’re always thinking about bills and stuff and I’m like, I don’t wanna pay an ambulance bill when I can walk into the hospital by myself.” –survivor
	4. Proximity to the hospital	“We didn’t think about 911 because we felt like by the time 911 came to us, we would probably be there… and it’s not all that far across town. Just a few miles to the hospital. We felt like we could get there with my condition.” –survivor
		“I don’t think it occurred to her. She just wanted to get me to the hospital. She was willing to drive me.” –survivor
	5. Distrust in healthcare providers	“I’ve always been afraid—I’ve always been afraid of doctors. I’ll just be right up front and honest with you, that is—even as a child.” –survivor
		“...usually with my experience, when you call 911, you get a whole bunch of questions and answers and back and forth... then finally, the ambulance, after we’ve all been standin’ around for what seem like 30 minutes.” –survivor
		“Even if they trust their doctor somewhat, they don’t trust hospitals. They really don’t. They’re just convinced that being in a hospital is gonna kill ‘em.” –caregiver
		“I may have [called 9-1-1] myself, but I wasn’t really thinking that way ‘cause you know how men are, we don’t like to go to the doctor or the hospital.” –survivor
	6. Concern of perceived overreacting	“I didn’t want to be the boy who cried wolf. I’d have symptoms like that three times a month. If I went to a doctor every time fingers or hands got numb, I’d constantly be in the hospital.” –survivor
		“I think a lot of people, especially men, but some women too… they’re really afraid of getting there and having it be nothing and having somebody tellin ‘em they came in for no reason, for not a serious reason” –caregiver

*Ordered by frequency of code counts.

### Facilitators to Calling 9-1-1 for Stroke

Rapid recognition or suspicion of stroke signs and symptoms was a common facilitator to calling 9-1-1. Prior lived experience with stroke, either through self, family, or acquaintance, was often accompanied by early recognition and feelings of fear or anxiety, prompting earlier care-seeking (Table 2). One interviewee expressed that due to experiencing a previous stroke in her mother, she knew that “once somebody has a stroke, they gotta get medical attention right away.” Many who called 9-1-1 were aware of the time-sensitive nature of stroke and the need to seek care immediately. Multiple participants mentioned previous exposure to stroke education from their doctor’s office and public education campaigns (e.g., billboards, public radio). Severe symptoms and their rapid onset prompted survivors and caregivers to recognize the need for immediate emergency medical care and led to calling 9-1-1.

Bystanders (sometimes the patient’s caregiver) were most frequently a spouse, adult child, friend, or neighbor and often strongly influenced the decision to call 9-1-1, even when the individual experiencing symptoms was hesitant. With initial denial of symptoms, one patient stated that he did not know “that this was a 9-1-1…that was [my daughter] just taking command of the situation.” In several instances when 9-1-1 was called early, an initial call was made to a family member or other trusted individual who was a healthcare professional (“called a friend of ours first, who’s a neuro ICU nurse”). This individual typically recognized or suspected a stroke and communicated the need to seek immediate emergency medical care.

### Barriers to Calling 9-1-1 for Stroke

Perceiving symptoms as not being severe was a common barrier to calling 9-1-1 (Table 2). Participants often described mild or ambiguous symptoms, such as weakness, numbness, or headache, and attributed them to alternative causes like stress, overexertion, or medication side effects. Even when aware of stroke warning signs, some did not believe their symptoms applied. Several expressed the belief they could “sleep it off” or take a “wait and see” approach. This perception was reinforced by a belief that EMS should be reserved for the most serious events. Participants frequently described calling 9-1-1 as appropriate only for life-threatening events, not “minor” concerns. Some also worried about being perceived as overreacting (like the “boy who cried wolf”), especially when alone and lacking bystanders to validate symptoms.

Financial concerns were another major barrier. The cost of ambulance transport, particularly for those without insurance, led many to avoid calling 9-1-1. Instead, patients and caregivers often opted for self-transport, believing it to be cheaper and sometimes faster, especially when the patient was ambulatory and the hospital was nearby. Rural participants, who self-transported more often than those in urban settings, cited the desire to drive rather than wait for an ambulance. Distrust of healthcare providers was also a barrier to calling 9-1-1 and seeking emergency medical care. Some participants described general distrust of physicians and hospitals and reluctance to seek care. This distrust appeared to be related to diverse reasons, including prior negative experience with EMS, gendered norms (“men don’t go to the hospital”), and longstanding fears of the medical system, sometimes dating back to childhood.

## Discussion

This qualitative study of a diverse sample of stroke survivors and caregivers from North Carolina found that prior stroke experience, knowledge of stroke signs, severe and rapid-onset symptoms, and the presence of a bystander were associated with timely 9-1-1 calls. On the other hand, participants who delayed or avoided calling 9-1-1 tended to report their symptoms as mild, believe that EMS was not warranted, and fear being perceived as overreacting. Financial concerns with ambulance and hospital costs and a general distrust of healthcare providers influenced participants’ decisions to seek immediate emergency medical care. These facilitators and barriers to calling 9-1-1 were observed across rural and non-rural settings.

The facilitators and barriers to calling 9-1-1 identified by our study for stroke could certainly apply to other severe conditions with sudden onset like myocardial infarction and traumatic injury. However, strokes pose unique challenges including often being painless and involving neurologic deficits that limit a person’s ability to recognize or communicate symptoms. For these reasons, we believe the role of bystanders in calling 9-1-1 is especially critical for stroke, and our study highlights their importance in promoting early recognition and action.

Despite our study sample’s demographic and geographic concentration, our findings are consistent with prior work showing the influence of stroke knowledge, symptom severity, and bystanders on early EMS activation.^10,23^ General issues related to healthcare costs, distrust of healthcare, and sociocultural and gender norms are less often reported as barriers to decision-making by stroke patients and caregivers. Potential high costs associated with the use of EMS are especially a public concern when it comes to ground ambulance bills, which are highly variable and notably not included in the No Surprises Act.^32^ Socioeconomic factors may influence individual decision-making in context-dependent ways. One study of stroke in an underserved, urban population observed that ambulances were used relatively more often because of the lack of other transport options. We found that distrust in healthcare was commonly longstanding and often a reflection of the entire healthcare system. Specific mistrust of EMS was influenced by past negative experiences. Our finding on the influence of masculinity in resisting or delaying emergency medical care for stroke symptoms requires further investigation. Female stroke patients in the ED have been observed to arrive by EMS more than male stroke patients.^4,33^ However, women tend to have greater awareness of stroke signs and symptoms and the need to seek care immediately, which may explain the gender differences in care-seeking behavior relatively more than gender norms.^34,35^

The role of social connections in stroke response appears complex. For some participants, early stroke recognition and calling 9-1-1 were accompanied by an initial call to or contact with a relative or friend working in a healthcare profession. Similarly, a German study found that stroke patients were more likely to present within 4.5 hours of symptom onset if the family member they initially called recognized the symptoms.^36^ However, in a mostly Black urban population in the United States, 75% of stroke patients interviewed called a relative or friend first and delayed calling 9-1-1 despite suspecting stroke. Neither study reported or commented on the professional identity of relatives/friends. In our study, participants who initially called a family/friend healthcare professional were encouraged to call 9-1-1 by this individual and were more likely to seek early EMS care. Prior research has shown that small and close-knit social networks are generally related to delays in seeking care for time-critical patients experiencing a heart attack or stroke.^37,38^ Based on our findings, the social environment should be treated as multi-faceted and warrants further research.

Our study sought to investigate the role of rurality on care-seeking during acute stroke. EMS is crucial for rural areas given the limited geographic access to stroke centers and the extra importance of early identification and triage of acute stroke.^39,40^ While the facilitators and barriers to calling 9-1-1 for stroke were consistent between rural and non-rural settings, self-transports occurred more often with rural strokes in our study. This observation is supported by prior studies showing that people in rural areas are less aware of the stroke signs and symptoms compared to those in urban/suburban settings.^41^ Resulting delays are especially concerning considering that rural patients presumably need to travel longer distances and are more likely to initially present at a non-stroke center.^15^

Future directions should focus on operationalizing these findings into experience-informed interventions that directly address the psychological and structural barriers identified. Public health messaging should extend stroke symptom education to address the fear of “overreacting,” framing 9-1-1 calls as a necessary precaution rather than a potential burden to the system. Additionally, effective interventions will need to consider socioeconomic factors, such as ambulance cost concerns and systemic distrust, perhaps through community-based advocacy or transparent EMS billing policies. Given the neurological barriers that often sideline the patient, future educational efforts should prioritize bystander activation and caregiver-focused training, equipping those most likely to witness a stroke with the agency to override patient denial. Finally, there is a critical opportunity for health systems and EMS agencies to co-design care-seeking protocols alongside patients and caregivers. Anchoring these recommendations in the lived experience of stroke survivors ensures that future interventions are not only clinically sound but are also culturally and emotionally resonant, ultimately shortening the bridge between symptom onset and emergency care.

## Limitations

Certain study limitations should be noted. Findings may be affected by recall error, as participants, particularly stroke survivors, may have had challenges remembering past experiences. We attempted to mitigate this by interviewing survivor-caregiver dyads in some cases. Our study of stroke survivors excluded the experiences of fatal strokes, possibly missing insights into promoting early care-seeking for the most severe cases. Although people not recognizing a stroke are the primary concern, interviewing non-stroke patients who believed they were having a stroke and sought care could reveal additional facilitators. These out-of-scope populations are important directions for future research. The sample was based on convenience recruitment from a single state with greater representation from central NC, limiting generalizability. Participants were all non-Hispanic and English-speaking, excluding the important perspectives of underrepresented groups. While we reached code saturation with no new codes emerging during the coding process, we may not have reached meaning saturation within certain themes due to our relatively homogenous sample.^29,30^ These limitations highlight the need for tempering generalizations beyond the characteristics of the study sample and for future research that includes more diverse populations from broader geographic regions.

## Conclusions

Through in-depth interviews of stroke survivors and caregivers from North Carolina across a range of contexts, this study identified barriers to calling 9-1-1, including perception of non-serious symptoms, fear of overreacting, and cost concerns. We recommend these barriers be addressed in stroke education tools and campaigns. Future research needs to design and test stroke care-seeking interventions that consider diverse clinical, sociodemographic, and community settings.

## Supplemental Material

Supplemental Material - Stroke Survivor and Caregiver Perspectives on Seeking Emergency Medical Care Supplemental Material for Stroke Survivor and Caregiver Perspectives on Seeking Emergency Medical Care by Emma N. Johnson, Lindsay E. Stewart, Edward C. Jauch, Jane H. Brice, and Mehul D. Patel in Journal of Patient Experience.

## Data Availability

Given the sensitive nature of the transcribed participant interviews, including participants’ protected health information, the authors do not plan to share these data publicly.*
