# Supplementary material for: Stroke Survivor and Caregiver Perspectives on Seeking Emergency Medical Care
Source: J Patient Exp. 2026 Jun 3;13:23743735261458028. doi: 10.1177/23743735261458028 (PMC13237252; doi:10.1177/23743735261458028)
Supplement: Supplemental Material - Stroke Survivor and Caregiver Perspectives on Seeking Emergency Medical Care [file sj-pdf-1-jpx-10.1177_23743735261458028.pdf]

Appendix 1. Interview Guides

Note: This is a semi-structured interview guide. Questions will vary based on the conversation but will be related to the topics included below.

Stroke Survivor Zoom Interview Guide
Interviewer Initials: __________
REDCap ID: ________

Interviewer Script

Hello <participant name>, my name is <interviewer name> from the Department of Emergency Medicine Research at UNC Chapel Hill. Thank you for taking the time to speak to me today about your experience with stroke care!
Ask if the person has any questions or concerns about the information provided in the Stroke Study Information page, and address/review accordingly.

Do you have any other questions for me about this research or your participation?

Would you like to participate in the interview for this study?
[YES] Great. Thank you!
[NO] No problem, thank you for your time!

Do you agree to be recorded?
[YES] Great. I'll start the recording now.
[NO] That's fine. We can proceed without the recording.

Interview Questions

Introduction:
First, I'd like to ask for some basic background information about your stroke.

1. How many strokes have you had in your lifetime?

2. What was the approximate date on which your most recent stroke occurred?

3. To your knowledge, what type of stroke did you have on that date?
<note: the two most common types of strokes are ischemic stroke - when blood vessels in the brain are blocked by blood clots, plaque, or other particles, and hemorrhagic stroke – when an artery in the brain ruptures or leaks blood.>

4. To which hospital did you go for your stroke care/treatment?

Priority Questions:
Now, I'm going to ask questions about your experience when you had your (more recent) stroke.

5. Think back to the day that your stroke occurred. Could you describe what you (or someone around you) noticed?
   <probe: What were your symptoms? facial, behavior, talking, breathing, moving> (1,2)
    a. What, if anything, did you do to deal with these symptoms? (2)
    b. What did the people around you (if there were any) do to help you? (2)

6. At the time, why did you think you were experiencing these symptoms?
    a. Can you describe how you felt about what was happening? / What was going through your mind? Did you know you were experiencing a stroke? (3)

7. Did you think you needed medical help? Why or why not? (3)
    a. At the time, how quickly did you think that you needed to be seen by a medical professional?

       <probe: How urgent or non-urgent did you think the situation was?> (4)

8. After your stroke symptoms were noticed, can you walk me through what happened next? (5,6)
    a. Did you (or someone around you) make a phone call for help?
    b. How long from the start of your symptoms did it take for you (or someone else) to take the next steps? Was there anything that caused delays?

9. How long after noticing your stroke symptoms did you (or someone else) call for help? Who was called? (3,5)
    a. **[IF 9-1-1 CALLED]→**Why did you (or someone else) call 9-1-1?
        i. How was your experience speaking with the 9-1-1 operator?
    b. **[IF 9-1-1 NOT CALLED]→**Why did you (or someone else) not call 9-1-1?

10. Can you talk (more) about why the decision was made to call / not to call for medical help? What are the reasons why you decided to call / not to call? (4)
    a. What concerns did you have, if any, about calling?
       <probe - offer only if needed: Some people are concerned about things like cost, contacting their physician, severity of their symptoms. Did any of these considerations come to your mind?>

[SKIP 11 IF EMS WAS NOT CALLED]

11. Please describe your experience with the first responders and EMS personnel. (7)

<probes: Who was the first to arrive on the scene (fire-fighters versus EMS)? What happened after they arrived? What was it like interacting with these responders/personnel?>

a. How did you feel about the ways that these personnel treated your stroke?l
b. What, if anything, do you wish had been different?
c. Was there anything that caused delays?

12. Did EMS discuss which hospital you were being taken to?

<probes: What sorts of things were discussed? How did you decide where to go? How long did you talk about this?>

[SKIP 13 IF EMS WAS CALLED]

13. Did you drive yourself (or someone else drive you) to the emergency room?

<probe: Where did you go if not to the ER?>

a. Can you talk about why you decided to drive to [insert location]?]
b. If not to the emergency room, can you share more about why you did not feel that you needed to go to the ER?
c. Was there anything that caused delays?

14. Is there anything else we haven't mentioned that played a role in how quickly you did or did not seek care?

<probe - offer only if needed: Some people are concerned about things like cost, contacting their physician, severity of their symptoms. Bystanders / other people around? Did any of these considerations come to your mind?>

Non-priority Questions:

Now, I'd like to ask some questions about your experience in the emergency room/department and the hospital.

15. Can you tell me about your experience when you arrived at the emergency room? (3)

a. How did you feel about the ways your clinicians (ER doctors, ER nurses, etc.) treated your stroke? (7)
   <probe: did you feel satisfied? dissatisfied? Why?>
b. What, if anything, do you wish had been different?

16. Can you tell me about your experience in the hospital?

a. How did you feel about the ways your hospital clinicians (doctors, nurses, neurologists, physical therapists, etc.) treated your stroke? (7)
b. What, if anything, do you wish had been different during your hospital stay?

17. How prepared or unprepared did you feel at the time of your discharge for your stroke care once back at home? (7)
    a. What, if anything, do you wish had been different during this transition? ***<probes: how supported or unsupported did you feel to handle your care? Did you feel that you were given the information you needed – why or why not?>***

Lastly, we have some final questions that will help us in designing and improving stroke education.

18. Based on your experience, what do you think is the most important information that people should know about strokes?
    a. What, if anything, didn't you know until after you had your stroke?
    b. What do you wish you, a caregiver, or a family member/friend, had known?

Additional Comments:

19. Is there something else you want to share or talk about that we did not cover?

Conclusion:

Thank you very much for your feedback, <participant name>! This has been extremely helpful. I am going to send you a follow-up email that will include a link to enter your information to receive the digital gift card for your participation today.

Additionally, at the end of study, we will write up a summary of general study findings (which will not include any participant identifiers or details) to share with participants. Would you like to receive this via email at study close?

In the meantime, please don't hesitate to reach out if any questions or issues come to mind. Thank you again. Goodbye.

1. Malek AM, Adams RJ, Debenham E, Boan AD, Kazley AS, Hyacinth HI, et al. Patient awareness and perception of stroke symptoms and the use of 911. J Stroke Cerebrovasc Dis. 2014 Oct;23(9):2362–71.

2. Wang PY, Tsao LI, Chen YW, Lo YT, Sun HL. "Hesitating and Puzzling": The Experiences and Decision Process of Acute Ischemic Stroke Patients with Prehospital Delay after the Onset of Symptoms. Healthcare (Basel). 2021 Aug 19;9(8):1061.

3. Bakke I, Lund CG, Carlsson M, Salvesen R, Normann B. Barriers to and facilitators for making emergency calls - a qualitative interview study of stroke patients and witnesses. J Stroke Cerebrovasc Dis. 2022 Oct;31(10):106734.

4. Schroeder EB, Rosamond WD, Morris DL, Evenson KR, Hinn AR. Determinants of Use of Emergency Medical Services in a Population With Stroke Symptoms. Stroke. 2000 Nov;31(11):2591–6.

5. Faiz KW, Sundseth A, Thommessen B, Rønning OM. Factors related to decision delay in acute stroke. J Stroke Cerebrovasc Dis. 2014 Mar;23(3):534–9.

6. Andersson J, Jakobsson S, Rejnö Å, Hansson P, Nielsen SJ, Björck L. Decision- Making in Seeking Emergency Care for Stroke Symptoms. Stroke: Vascular and Interventional Neurology. 2022 Nov;2(6):e000376.

7. Perry C, Papachristou I, Ramsay AIG, Boaden RJ, McKevitt C, Turner SJ, et al. Patient experience of centralized acute stroke care pathways. Health Expect. 2018 Oct;21(5):909–18.

Note: This is a semi-structured interview guide. Questions will vary based on the conversation but will be related to the topics included below.

Stroke Caregiver Zoom Interview Guide
Interviewer Initials: __________
REDCap ID: ________

Interviewer Script

Hello <participant name>, my name is <interviewer name> from the Department of Emergency Medicine Research at UNC Chapel Hill. Thank you for taking the time to speak to me today about your experience with stroke care!
Ask if the person has any questions or concerns about the information provided in the Stroke Study Information page, and address/review accordingly.

Do you have any other questions for me about this research or your participation?

Would you like to participate in the interview for this study?
[YES] Great. Thank you!
[NO] No problem, thank you for your time!

Do you agree to be recorded?
[YES] Great. I'll start the recording now.
[NO] That's fine. We can proceed without the recording.

Interview Questions

Introduction:
First, I'd like to ask for some basic background information about your [insert relationship]'s stroke.

1. How many strokes has your [insert relationship] had in his/her lifetime?

2. What was the approximate date on which your [insert relationship]'s most recent stroke occurred?

3. To your knowledge, what type of stroke did your [insert relationship] have on that date? ***<note: the two most common types of strokes are ischemic stroke - when blood vessels in the brain are blocked by blood clots, plaque, or other particles, and hemorrhagic stroke – when an artery in the brain ruptures or leaks blood.>***

4. To which hospital did your [insert relationship] go for his/her stroke care/treatment?

Priority Questions:
Now, I'm going to ask questions about your experience when your [insert relationship] had his/her (more recent) stroke.

5. Think back to the day that the stroke occurred. Could you describe what you noticed?

   <probe: What were your [insert relationship]'s symptoms? facial, behavior, talking, breathing, moving> (1,2)
   a. What, if anything, did you do to help your [insert relationship] deal with these symptoms? (1)
   b. Were there other people around? What, if anything, did they do to help? (1)

6. At the time, why did you think your [insert relationship] was experiencing these symptoms?
   a. Can you describe how you felt about what was happening? / What was going through your mind? Did you know that your [insert relationship] was experiencing a stroke? (3)

7. Did you think that your [insert relationship] needed medical help? Why or why not? (3)
   a. At the time, how quickly did you think that your [insert relationship] needed to be seen by a medical professional?

   <probe: how urgent or non-urgent did you think the situation was?> (4)

8. After your [insert relationship]'s stroke symptoms were noticed, can you walk me through what happened next? (5,6)
   a. Did you (or someone else) make a phone call for help?
   b. How long from the start of your symptoms did it take for you (or someone else) to take the next steps? Was there anything that caused delays?

[SKIP 9 IF NO CALL FOR HELP MADE]

9. How long after noticing the stroke symptoms did you (or someone else) call for help? Who was called? (3,5)
   a. **[IF 9-1-1 CALLED]**→Why did you (or someone else) call 9-1-1?
      i. How was your experience speaking with the 9-1-1 operator?
   b. **[IF 9-1-1 NOT CALLED]** →Why did you (or someone else) not call 9-1-1?

10. Can you talk (more) about why the decision was made to call / not to call for medical help? What are the reasons why you decided to call / not to call? (4)
    a. What concerns did you have, if any, about calling?

       <probe - offer only if needed: Some people are concerned about things like cost, contacting their physician, severity of their symptoms. Did any of these considerations come to your mind?>

[SKIP 11 IF EMS WAS NOT CALLED]

11. Please describe your experience with the first responders and EMS personnel. (7)

    <probes: Who was the first to arrive on the scene (fire-fighters versus EMS)? What happened after EMS arrived? What was it like interacting with these responders/personnel?>

    a. How did you feel about the ways these personnel treated your [insert relationship]'s stroke?
    b. What, if anything, do you wish had been different?
    c. Was there anything that caused delays?

12. Did EMS discuss which hospital your [insert relationship] was being taken to?

    <probes: What sorts of things were discussed? How did you make a decision about where to go? How long did you talk about this?>

[SKIP 13 IF EMS WAS CALLED]

13. Did your [insert relationship] drive him/herself or have you/someone else drive him/her to the emergency room?

    <probe: Where did he/she go if not to the ER?>

    a. Can you talk about why you (or someone else) decided to drive your [insert relationship] to [insert location]?
    b. If not to the emergency room, can you share more about why your [insert relationship] (and/or you) did not feel that he/she needed to go to the ER?
    c. Was there anything that caused delays?

14. Is there anything else we haven't mentioned that played a role in how quickly you did or did not seek care?

    <probe - offer only if needed: Some people are concerned about things like cost, contacting their physician, severity of their symptoms. Bystanders / other people around? Did any of these considerations come to your mind?>

Non-priority Questions:

Now, I'd like to ask some questions about your experience in the emergency room/department and the hospital. At any point, were you with your [insert relationship] in the emergency department? In the hospital room?

[SKIP 15 IF RESPONDENT NOT PRESENT IN ED]

15. Can you tell me about your experience when you and your [insert relationship] arrived at the emergency room? (3)

    a. How did you feel about the ways the clinicians (ER doctors, ER nurses, etc.) treated your [insert relationship]'s stroke? (7)

       <probe: did you feel satisfied? dissatisfied? Why?>

b. What, if anything, do you wish had been different?

[SKIP 16 IF RESPONDENT NOT PRESENT IN HOSPITAL]

16. Can you tell me about your experience in the hospital with your [insert relationship]?
    a. How did you feel about the ways the hospital clinicians (doctors, nurses, neurologists, physical therapists, etc.) treated your [insert relationship]'s stroke? (7)
    b. What, if anything, do you wish had been different during your [insert relationship]'s hospital stay?

17. How prepared or unprepared did you feel at the time of your [insert relationship]'s hospital discharge for his/her stroke care once back at home? (7)
    a. What, if anything, do you wish had been different during this transition?

<probes: how supported or unsupported did you feel to handle his/her care? Did you feel that you were given the information you needed – why or why not?>

Lastly, we have some final questions that will help us in designing and improving stroke education.

18. Based on your experience, what do you think is the most important information that people should know about strokes?
    a. What, if anything, didn't you know until after your [insert relationship] had his/her stroke?
    b. What do you wish you, your [insert relationship], or another family member/friend, had known?

Additional Comments:

19. Is there something else you want to share or talk about that we did not cover?

Conclusion:

Thank you very much for your feedback, <participant name>! This has been extremely helpful. I am going to send you a follow-up email that will include a link to enter your information to receive the digital gift card for your participation today.

Additionally, at the end of study, we will write up a summary of general study findings (which will not include any participant identifiers or details) to share with participants. Would you like to receive this via email at study close?

In the meantime, please don't hesitate to reach out if any questions or issues come to mind. Thank you again. Goodbye.

1. Wang PY, Tsao LI, Chen YW, Lo YT, Sun HL. “Hesitating and Puzzling”: The Experiences and Decision Process of Acute Ischemic Stroke Patients with Prehospital Delay after the Onset of Symptoms. Healthc Basel Switz. 2021 Aug 19;9(8):1061.

2. Malek AM, Adams RJ, Debenham E, Boan AD, Kazley AS, Hyacinth HI, et al. Patient awareness and perception of stroke symptoms and the use of 911. J Stroke Cerebrovasc Dis Off J Natl Stroke Assoc. 2014 Oct;23(9):2362–71.

3. Bakke I, Lund CG, Carlsson M, Salvesen R, Normann B. Barriers to and facilitators for making emergency calls - a qualitative interview study of stroke patients and witnesses. J Stroke Cerebrovasc Dis Off J Natl Stroke Assoc. 2022 Oct;31(10):106734.

4. Schroeder EB, Rosamond WD, Morris DL, Evenson KR, Hinn AR. Determinants of Use of Emergency Medical Services in a Population With Stroke Symptoms. Stroke. 2000 Nov;31(11):2591–6.

5. Faiz KW, Sundseth A, Thommessen B, Rønning OM. Factors related to decision delay in acute stroke. J Stroke Cerebrovasc Dis Off J Natl Stroke Assoc. 2014 Mar;23(3):534–9.

6. Andersson J, Jakobsson S, Rejnö Å, Hansson P, Nielsen SJ, Björck L. Decision-Making in Seeking Emergency Care for Stroke Symptoms. Stroke Vasc Interv Neurol. 2022 Nov;2(6):e000376.

7. Perry C, Papachristou I, Ramsay AIG, Boaden RJ, McKevitt C, Turner SJ, et al. Patient experience of centralized acute stroke care pathways. Health Expect Int J Public Particip Health Care Health Policy. 2018 Oct;21(5):909–18.

Appendix 2. Final Codebook

Code Name	Type	Definition	Subcodes
Background - stroke experience	Deductive	Apply this code when the interviewee discusses their background experience in acute stroke care, whether that be as a stroke survivor or carepartner (e.g., number of strokes experienced, type of stroke, treatment destination, etc.)	*Unsure/unreliable* : Apply this subcode when the patient is unsure of previous strokes or is a poor historian<br><br>*Two+ strokes* : Apply this subcode when the patient has had two or more strokes<br><br>*First stroke* : Apply this subcode when the interview indicates that this is the patient's first stroke
Process - symptoms	Deductive	Apply this code when the interviewee discusses what they noticed in terms of symptoms during the survivor's most recent stroke	*Timeframe to onset of symptoms* : Apply this subcode when interviewee discusses symptom onset, including the following subcodes:<br>*Progressive - 1+ weeks* : Apply this subcode when the symptoms progressed over the course of one or more weeks<br>*Progressive - several days* : Apply this subcode when the symptoms progressed over the course of several days<br>*Progressive - same day* : Apply this subcode when the symptoms progressed the same day<br>*Instant* : Apply this subcode when the symptoms were instantaneous<br><br>Apply the following subcodes when the interviewee mentions these respective symptoms:<br><br>*Unresponsive/loss of consciousness/convulsions*<br><br>*Disorientation/delirium*<br><br>*Loss of temperature sensitivity*<br><br>*Hearing loss/earaches*<br><br>*Defecation/urination/vomiting*<br><br>*Headache/lightheaded/dizziness*<br><br>*Vision issues*<br><br>*Difficulty swallowing*<br><br>*One-sided weakness/numbmess/paralysis*<br><br>*Facial droop/drooling*<br><br>*Lack of coordination/balance*<br><br>*Broken speech/slurring/inability to speak*
Process - help seeking	Deductive	Apply this code when the interviewee discusses from where they sought help, if at all	Apply the following subcodes when the interviewee discusses their first point of contact (or lack thereof) when seeking help for stroke symptoms:<br><br>*Went to urgent care (UC) first*<br><br>*Patient in long term care (LTC) facility at time of stroke*<br><br>*Patient inpatient at time of stroke*<br><br>*Self-transport to emergency department (ED)*<br><br>*Couldn't reach phone to make a call*<br><br>*Health care provider (HCP) friend/family/neighbor called first*<br><br>*General practicioner (GP) called first*<br><br>*9-1-1 called first*

Process - 9-1-1	Deductive	Apply this code when the interviewee discusses interactions with the 9-1-1 operator, if applicable	*Timeline from start of symptoms to call* : Apply this subcode when the interviewee discusses their timeline to calling 9-1-1, including the following subcodes:<br>*Immediate (<1-2 minutes)*<br>*5-7 minutes*<br>*45 minutes-1hour*<br>*>1 hour*<br><br>Apply the following subcodes when the interviewee discusses their specific interactions with the 9-1-1 operator or reasons why the interviewee called 9-1-1:<br><br>*Caller unfamiliar with area (located via GPS)*<br><br>*Call to HCP friend preceded 9-1-1 call*<br><br>*Call switched to multiple operators to locate the correct county*<br><br>*Call made due to outside influence (caller didn't recognize as stroke)*<br><br>*Call made against patient's judgement/requests*<br><br>*Call handled by rehab/LTC facility*<br><br>*Call made/help arrived quickly*<br><br>*Coordinating details with dispatch (how to enter the house/where to go upon entering)*<br><br>*Operator spoke to pt to assess slurred speech*<br><br>*Operator remained on line until Emergency Medical System (EMS) arrival*<br><br>*Operator provided reassurance*<br><br>*Preliminary stroke screening by operator*
Process - EMS	Deductive	Apply this code when the interviewee discusses interactions with EMS on-scene and during transport processes, if applicable (e.g., identification/screening, on-scene management, triage and transport, etc.)	
Process - transfer of care	Deductive	Apply this code when the interviewee discusses transferring care of patient to ED or other care provider ("hand off" process), if applicable	
Process - self transport	Deductive	Apply this code when the interviewee discusses any aspect of self-transport to seek care, if applicable (e.g., where did they go, why did they go there, etc.)	Apply the following subcodes when the interviewee discusses reasons for self-transport:<br><br>*Previous mild stroke (thought symptoms would be similar)*<br><br>*Knew of need to get to hospital quickly*<br><br>*Advised by HCP to go to ER (not specifically to call 9-1-1)*<br><br>*Close proximity to hospital*<br><br>*Didn't trust EMS to arrive/transport quickly*<br><br>*Didn't want to call 9-1-1*<br><br>*Didn't think to call 9-1-1 (caregiver capable of transporting patient)*
Process - ER	Deductive	Apply this code when the interviewee discusses interactions with clinicians in the Emergency Room (ER), if applicable (e.g., what happened upon arrival at the ER))	
Process - hospital	Deductive	Apply this code with the interviewee discusses interactions with clinicians in the hospital, if applicable (e.g., what happened during their hospital stay)	
Process - discharge	Deductive	Apply this code when the interviewee discusses interactions with clinicians at the time of discharge (e.g., what discharge instructions were they given, what happened at the time of	
Perspectives - symptoms	Deductive	Apply this subcode when the interviewee discusses their perspectives regarding symptoms	*Considered stroke, but didn t think symptoms matched* : Apply this subcode when the interviewee was aware of stroke symptoms and considered stroke but didn't think their

			symptoms matched typical stroke symptoms
Perspectives - help seeking	Deductive	Apply this code when the interviewee discusses their decision making process for seeking help (or not seeking help), specifically who to call for help and why	*Called 9-1-1* : Apply this subcode when the interviewee mentions calling 9-1-1, including the following subcodes describing why:<br>*Thought they could wait/sleep it off* : Apply this subcode when the interviewee describes how they waited and subsequently called 9-1-1 due to symptoms getting more severe.<br>*Wanted to drive, but couldn't get pt in the car* : Apply this subcode when interviewee describes wanting to drive, but was unable to transport the patient themselves.<br>*Scared* : Apply this subcode when the interviewee describes how being scared influenced calling 9-1-1.<br>*Wanted to drive, but symptoms became too severe* : Apply this subcode when the interviewee discusses wanting to drive, but symptoms became too severe.<br>*Bystanders/HCP friends influenced perception of severity* : Apply this subcode when bystanders (particularly those who were HCP) influenced the survivor's perception of severity.<br>*Severe symptoms/knew something was wrong* : Apply this subcode when the interviewee knew something was wrong due to severe symptoms.<br>*Background stroke knowledge (similar symptoms at separate stroke incidence/certain of stroke)* : Apply this subcode when the interviewee had background knowledge and were aware of stroke due to prior experience.<br>*Needed help urgently* : Apply this subcode when the interviewee discusses needing help urgently.<br><br>*Didn't call 9-1-1:* Apply this subcode when the interviewee mentions calling 9-1-1, including the following subcodes describing why:<br>*Close proximity to the hospital (thought they could transport more quickly themselves)* : Apply this subcode when the interviewee thought self-transport would be faster than EMS due to close proximity to the hospital.<br>*Wanted to avoid hospital/ambulance* : Apply this subcode when the interviewee discusses wanting to avoid the hospital or transportation via ambulance.<br>*Didn't think symptoms to be fatal/life threatening* : Apply this subcode when the interviewee believed their symptoms weren't severe enough to call 9-1-1.<br>*"Holding pattern" to see if symptoms worsened* : Apply this subcode when the interviewee discusses waiting to see if symptoms worsened.<br>*Distrust* : Apply this subcode when the interviewee discusses avoiding EMS due to distrust of medical providers.
Perspectives - concerns about seeking medical attention	Deductive	Apply this code when interviewee discusses any concerns by the patient or others about seeking medical attention	*Being perceived as overreacting* : Apply this subcode when the interviewee discusses concerns regarding being perceived as overreacting by medical professionals.<br><br>*Fear of hospitals* : Apply this subcode when the interviewee is concerned due to fear of hospitals.<br><br>*Embarassment/not wanting to alert the neighbors* : Apply this subcode when the interviewee is hesitant to call 9-1-1 out of embarassment or not wanting to alert their neighbors.<br><br>*Concerned patient wouldn't go with EMS* : Apply this subcode when the interviewee is concerned that the patient wouldn't go with EMS.<br><br>*Cost* : Apply this subcode when the interviewee discusses cost as a concern.<br><br>*Concern for patient's health* : Apply this subcode when the interviewee discusses concern for the patient's health.<br><br>*No concerns* : Apply this subcode when the interviewee endorses no concerns about seeking medical attention.

Perspectives - 9-1-1	Deductive	Apply this code with the interviewee discusses their personal experience speaking with the 9-1-1 operator, if applicable (e.g., how did they feel about the way the operators treated the stroke)	Apply the following subcodes when the interviewee discusses their experiences and perceptions of 9-1-1:<br><br>*Knowledgeable/helpful operator*<br><br>*Comforting/calm operator*<br><br>*Positive experience*
Perspectives - EMS	Deductive	Apply this code when the interviewee discusses their personal experience interacting with EMS, if applicable (e.g., how did they feel about the way EMS treated the stroke, is there anything they wish would have been different)	
Perspectives - ER	Deductive	Apply this code with the interviewee discusses their personal experience in the ER, if applicable (e.g., how did they feel about the way their clinicians treated the stroke, is there anything they wish would have been different)	
Perspectives - hospital	Deductive	Apply this code with the interviewee discusses personal experience in the hospital, if applicable (e.g., how did they feel about the way their clinicians treated the stroke, is there anything they wish would have been different)	
Perspectives - discharge	Deductive	Apply this code when the interviewee discusses their personal experience during discharge (e.g., how did they feel about the way their clinicians prepared them for discharge, is there anything they wish would have been different)	
Delays	Deductive	Apply this code when the interviewee discusses anything that may have played a role in how quickly they did or did not seek and/or receive	
Stroke education	Deductive	Apply this code when the interviewee discusses their views on stroke education and what the most important things for the public to know about stroke are	
Care - facilitators	Deductive	Apply this code when the interviewee discusses what worked well in the processes used to care for the patient's acute stroke	
Care - barriers	Deductive	Apply this code when the interviewee discusses what did not work well in the processes used to care for the patient's acute stroke	

Figure S1. Pathways of EMS Activation for Stroke Recognition (19 distinct stroke events) (panel a) and EMS Activation Without Stroke Recognition (15 distinct stroke events) (panel b)

(a)

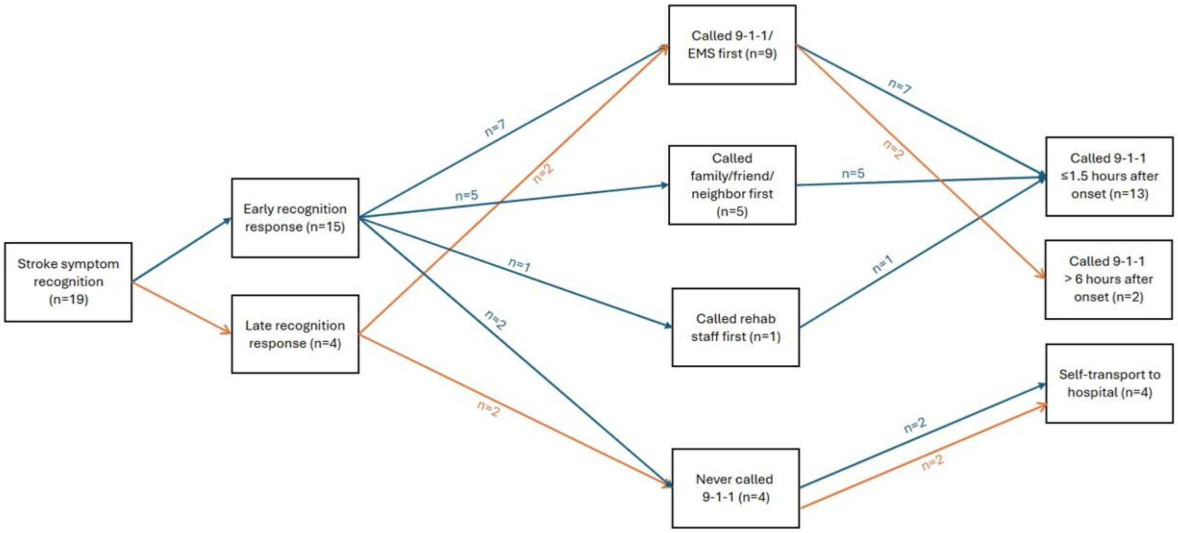

(b)

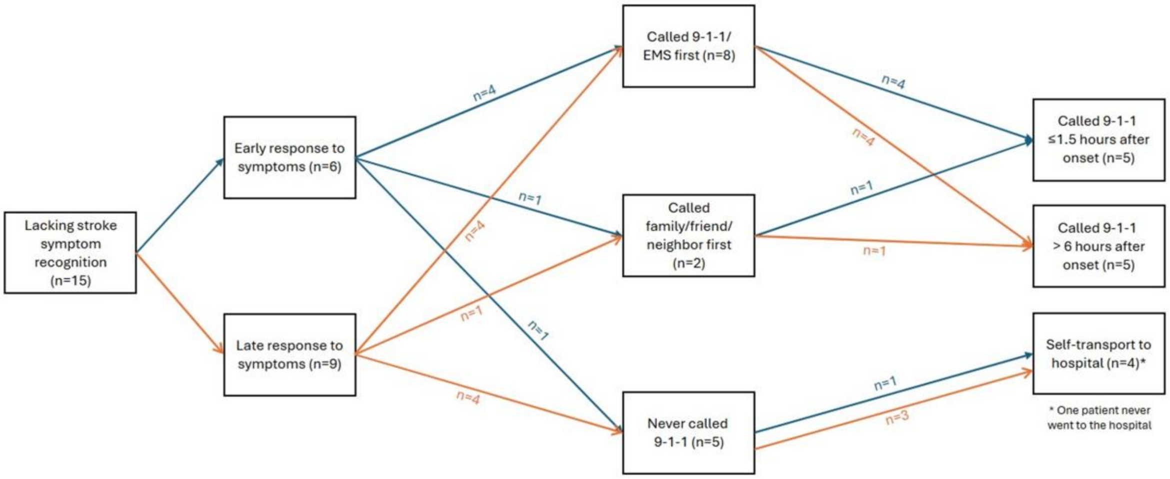

Early call is within 1.5 hours after symptom onset

Late call is 6 hours or greater after symptom onset
